# Supplementary material for: Circulating microbial content in myeloid malignancy patients is associated with disease subtypes and patient outcomes
Source: Nat Commun. 2022 Feb 24;13:1038. doi: 10.1038/s41467-022-28678-x (PMC8873459; doi:10.1038/s41467-022-28678-x)
Supplement: Supplementary file 1 — Supplementary Information [file 41467_2022_28678_MOESM1_ESM.pdf]

**<u>SUPPLEMENTARY NOTE</u>: ASSOCIATIONS REMAIN VALID DESPITE TEMPORAL CHANGES IN MICROBIAL CONTENT**

As shown in the Supplementary Figure 1 below, the t-SNE plot based on the relative abundances across all microbial genera shows some apparent grouping by date of processing, roughly into four time periods: September 2005 - January 2011, February 2011 - November 2011, December 2011 - February 2013, and March 2013 - February 2017.

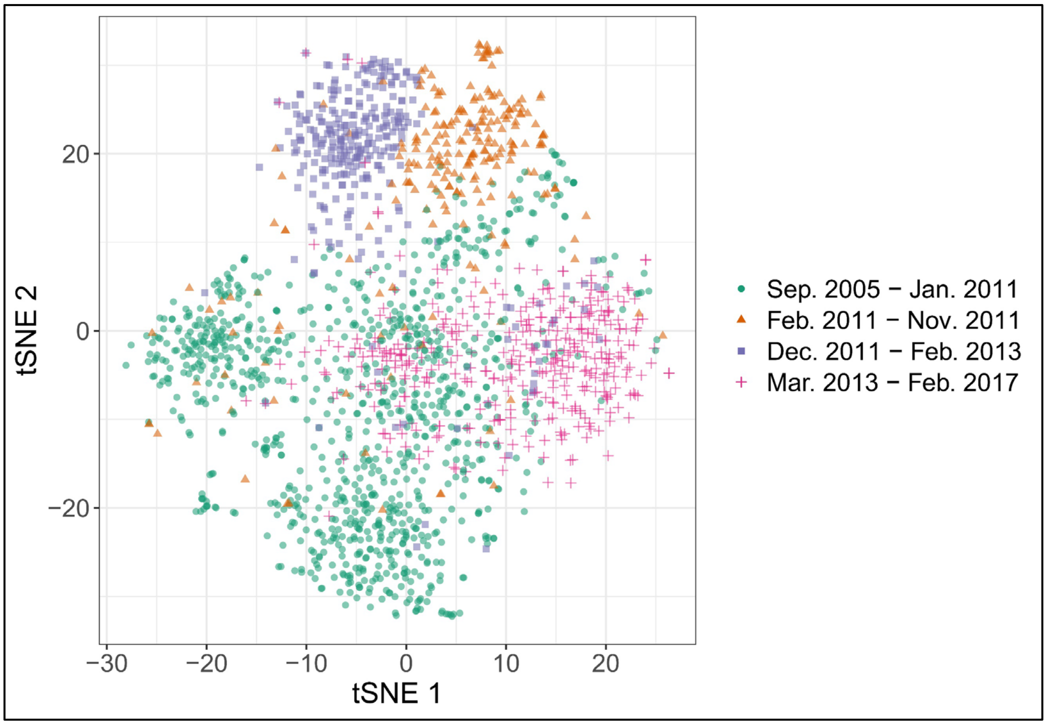


**<u>Supplementary Figure 1:</u> tSNE coordinates of individual patient samples colored by processing date.**

To ensure that these temporal differences did not lead to artifactual results, in this Supplementary Note we re-perform the statistical analyses presented in the Results section of the main text, this time controlling for membership in these four time periods. Controlling for time period entails either adjusting for time period in the statistical model (where possible) or re-performing the analysis within each time period separately. As we show here, the conclusions remain valid even while accounting for temporal effects, demonstrating that the temporal effects are not confounders in our analyses.

### Clustering by disease subtype in both tSNE and principal coordinate space

As shown in Figure 1b of the main text, MDS patients cluster together. The substantial majority (467 of 640, 73%) of MDS patients fall within the first (September 2005 – January 2011) time period shown in the Figure above. To ensure that the clustering observed in Figure 1b is not an artifact of the temporal clustering, we recomputed the t-SNE coordinates for patient samples from the September 2005 – January 2011 time period. As shown in Supplementary Figure 2 below, even within this time period the MDS patients show strong clustering. For instance, 226 of 467 MDS patients (48%) in this time period have second t-SNE coordinates larger than 10, while only 10 of 494 (2%) non-MDS patients do ($P < 2.2 \times 10^{-16}$).

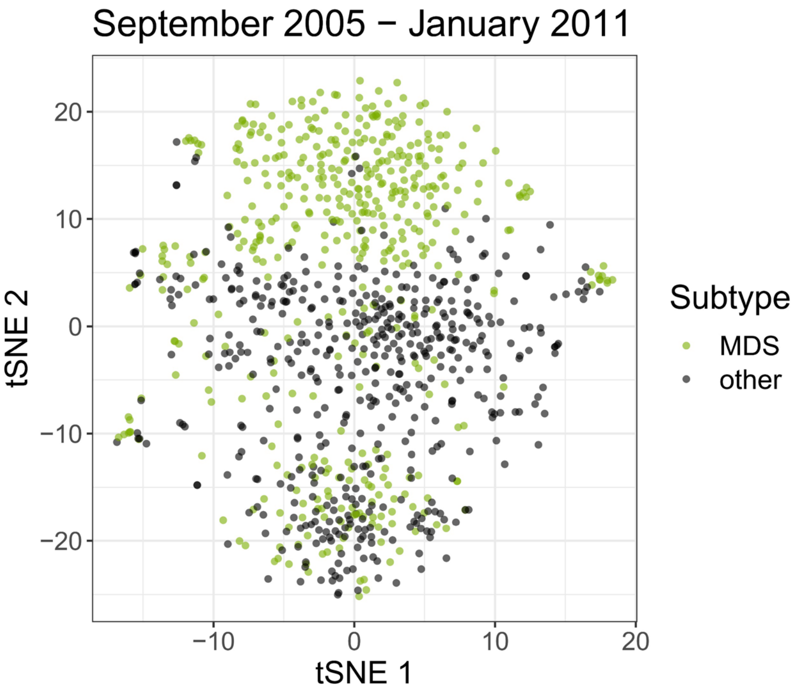


**<u>Supplementary Figure 2:</u> tSNE coordinates of individual patient samples processed September 2005-January 2011 colored by MDS status.**

Next, the main text reports an association (Figure 1f; chi-squared test $P < 2.2 \times 10^{-16}$) between patient disease subtypes and principal coordinate clusters. Testing for association between subtype and cluster membership *within* each of the four time periods yields chi-squared P-values of $8.2 \times 10^{-41}$, $2.8 \times 10^{-7}$, 0.047, and 0.158 for the first, second, third, and fourth time periods, respectively. Since all but the last time period showed at least nominal significance, it is unlikely that the observed association between subtype and principal coordinate cluster was confounded by temporal factors.

### Cluster association with karyotypic features

Figure 1g in the main text demonstrates differences in prevalence of karyotypic features among the principal coordinate clusters. The P-values in the figure are calculated using logistic regression. We can adjust for time period by adding binary indicator variables to the logistic regression model, as follows:

$$\text{logit}(p) = \beta_0 + \beta_1 \times I(\text{September 2005-January 2011}) + \beta_2 \times I(\text{February 2011-November 2011}) + \beta_3 \times I(\text{December 2011-February 2013}) + \beta_4 \times \text{PCo\_cluster} \quad (1)$$

where $p$ = probability of the karyotypic feature, PCo_cluster is a categorical variable indicating patient membership in one of the four principal coordinate clusters, and $I(x)$ is the indicator function/dummy variable

$$I(x) = \begin{cases} 1 \text{ if the patient was processed in time period } x \\ 0 \text{ otherwise} \end{cases} \quad (2)$$

*P*-values for the logistic regression model are computed using ANOVA. Without controlling for the time periods, the P-values (as shown in Figure 1g) are 0.00096, $1.83 \times 10^{-6}$, and 0.023 for associations between cluster and complex karyotype, normal karyotype, and trisomy 8, respectively. After adjusting for time period, these P-values become 0.00019, $7.7 \times 10^{-5}$, and 0.015, respectively. The associations remain significant, suggesting that the temporal changes are not confounders here.

### Viral involvement in MDS

In the main text, we report that MDS patients show the highest viral prevalence (logistic regression $P = 2.51 \times 10^{-5}$) and highest viral burden (Wilcoxon $P = 1.12 \times 10^{-8}$) among the four disease subtypes. The former still holds true when the logistic regression model is adjusted for time period in a manner similar to that shown in the logistic regression equation above ($P = 2.81 \times 10^{-5}$), and indeed the higher viral burden in MDS holds true within each of the four time period groups as shown in Supplementary Figure 3 below (although not all are statistically significant likely owing to smaller sample sizes, they all show higher median burden in MDS), further demonstrating that temporal change is not a confounder here:

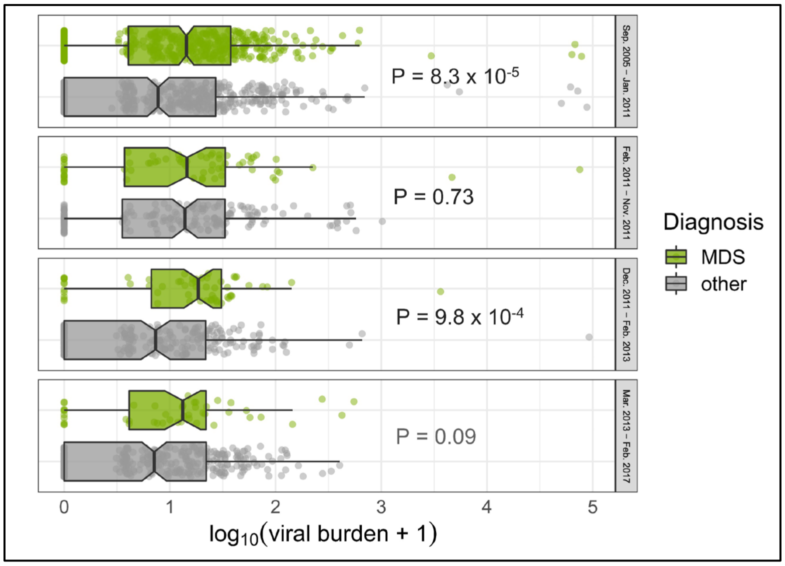


**<u>Supplementary Figure 3:</u> Viral burden, within each time period, stratified by MDS status.**

In MDS patients, worse overall survival was associated with EBV presence as reported in the main text (Figure 2d; age-adjusted Cox proportional hazards test HR 1.32, 95% CI 1.04 – 1.67, *P* = 0.022). When further adjusting for time period by adding terms into the Cox model (as in the logistic regression model above), we found a very similar association (HR 1.31, 95% CI: 1.03 - 1.67, *P* = 0.025).

We also reported in the main text that EBV presence/absence stratified the patients with “Low” IPSS-R risk scores to resemble the Intermediate/Very Low categories, respectively, with regard to survival. Specifically, as expected, individuals with IPSS-R Low had better survival than those with IPSS-R Intermediate (age-adjusted Cox P = 0.002) and worse survival than those with IPSS-R Very Low (age-adjusted Cox P = 0.098). However, when IPSS-R Low individuals are stratified by presence/absence of EBV, their survival is statistically indistinguishable from IPSS-R Intermediate/Very Low patients, respectively (P = 0.328 and 0.299, respectively). When adjusting

the model further for time period as above, the P-values remain similar (unstratified Low vs. Intermediate P = 0.008; unstratified Low vs. Very Low P = 0.082; Low w/ EBV vs. Intermediate P = 0.46; Low w/o EBV vs. Very Low P = 0.28).

It follows from these analyses that temporal changes are not a confounder in the observed role of EBV in MDS.

## Higher fungal detection in MDS

The main text reported higher prevalence of detected fungal reads among MDS patients (logistic regression $P$ = 0.00586). When adjusting for time period as above, the association remains significant ($P$ = 0.00143).

## Relationships between disease subtypes and bacterial relative abundance/diversity

We report overall association between disease subtypes and Proteobacteria relative abundance in the main text (age-adjusted ANOVA $P = 7.1 \times 10^{-7}$). If we further adjust for time period as above, the result remains significant ($P$ = 0.0087). For AML in particular, we found the highest Proteobacteria relative abundance (Wilcoxon $P < 2.2 \times 10^{-16}$). As shown in Supplementary Figure 4 below, higher Proteobacteria relative abundance in AML is consistent within each time period:

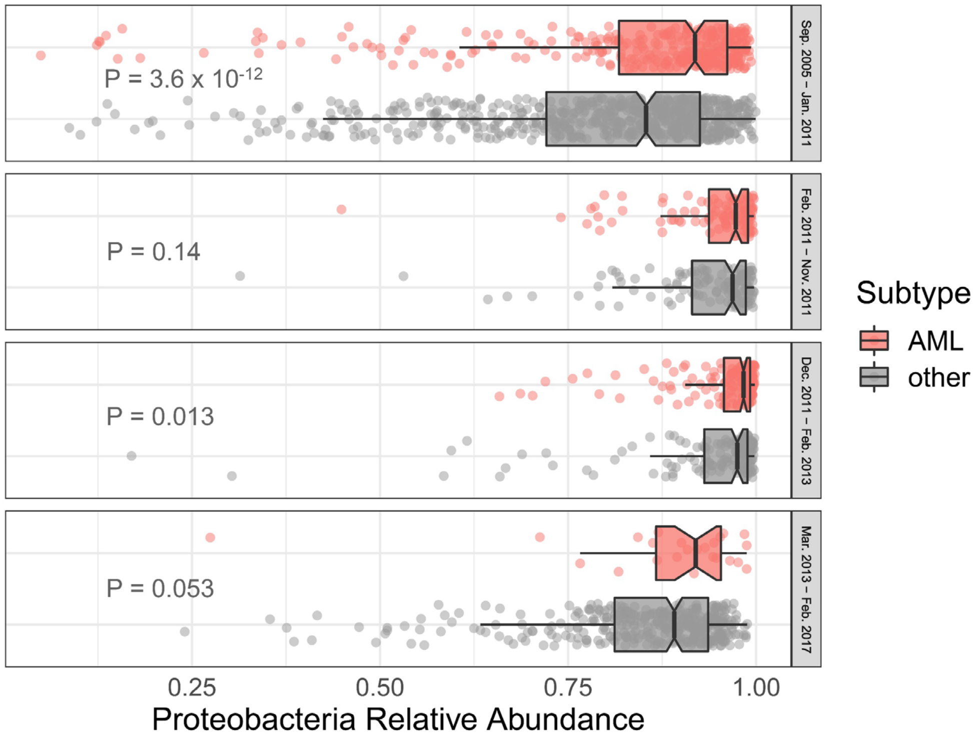


<u>Supplementary Figure 4:</u> **Proteobacteria relative abundance, within each time period, stratified by AML status.**

It was also shown in the main text that AML patients had the lowest α-diversity and richness (Figures 3c and 3e). To control for time period, for each taxonomic level we fit a logistic regression

model, modeling probability of (binary) AML status as a function of α-diversity, while controlling for age and time period. Specifically, the model is:

$$\text{logit}(p) = \beta_0 + \beta_1 \times I(\text{September 2005-January 2011}) + \beta_2 \times I(\text{February 2011-November 2011}) + \beta_3 \times I(\text{December 2011-February 2013}) + \beta_4 \times \text{age} + \beta_5 \times \alpha\text{-diversity} \quad (3)$$

where $p$ = probability of AML and $I(x)$ is the indicator function/dummy variable for time period as above. We also fit the model controlling only for age (i.e. omitting the $\beta_1$, $\beta_2$, and $\beta_3$ terms). As can be seen in Supplementary Figure 5 below, the coefficient estimates are negative for the α-diversity terms whether or not we control for time period, indicating that AML subtype consistently tracks with lower α-diversity (and is usually statistically significant, as indicated by the P-values accompanying each test, shown in figure) when controlling for time period:

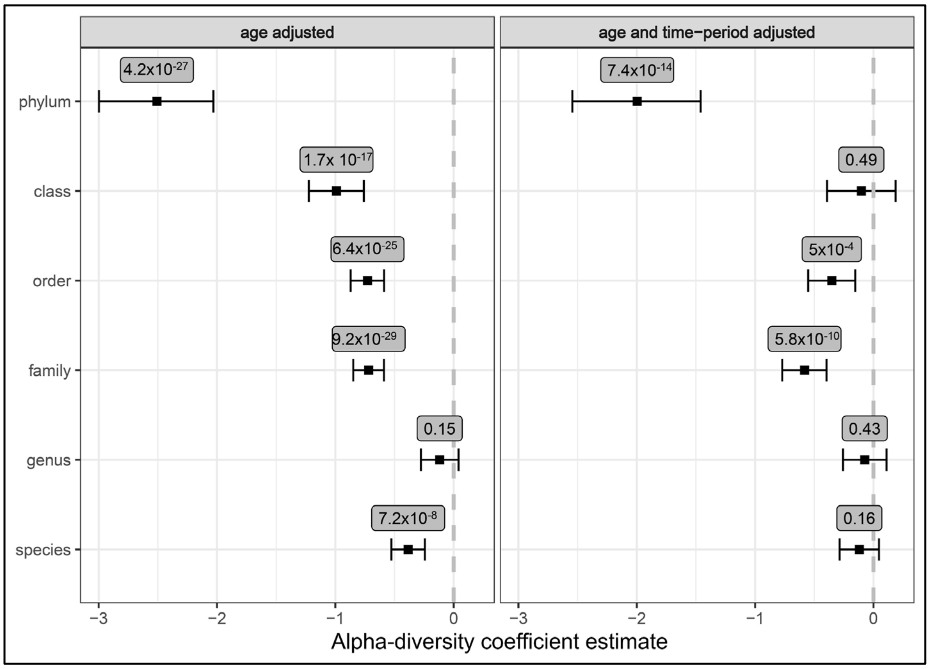


**<u>Supplementary Figure 5:</u> Coefficient estimates for α-diversity term in Supplementary Equation (3), both with (right panel) and without (left panel) time-period adjustment.**

With regard to richness, it can be seen from Supplementary Figure 6 below that the lower richness we observe across the entire cohort is largely consistent within time periods (although the numbers of AML samples in the last time period are admittedly too small ($n$ = 24) to observe much difference).

Taken together, these results indicate that our observed relationships between disease subtype and bacterial relative abundance/diversity are not confounded by temporal factors.

### Machine learning disease subtype classifiers

In the main text we demonstrate the ability of random forest-based classifiers to distinguish among disease subtypes based on bacterial content. To demonstrate that these classifiers were not affected by temporal changes in microbial content, we show here that classification models still perform substantially better than chance when trained and validated *within* each of the four time periods (see Supplementary Table 1 below). The performances of the within-time period

classifiers, as measured by AUROC, are not as strong as for the entire cohort, but this is to be expected since the numbers of patient training samples are much smaller within each time period, leading to a less-accurate classifier. Nonetheless, all of the AUROC estimates are above 0.5 (chance), and nearly all of the 95% confidence intervals are above 0.5, indicating that the temporal effects do not drive the subtype signal from microbial content.

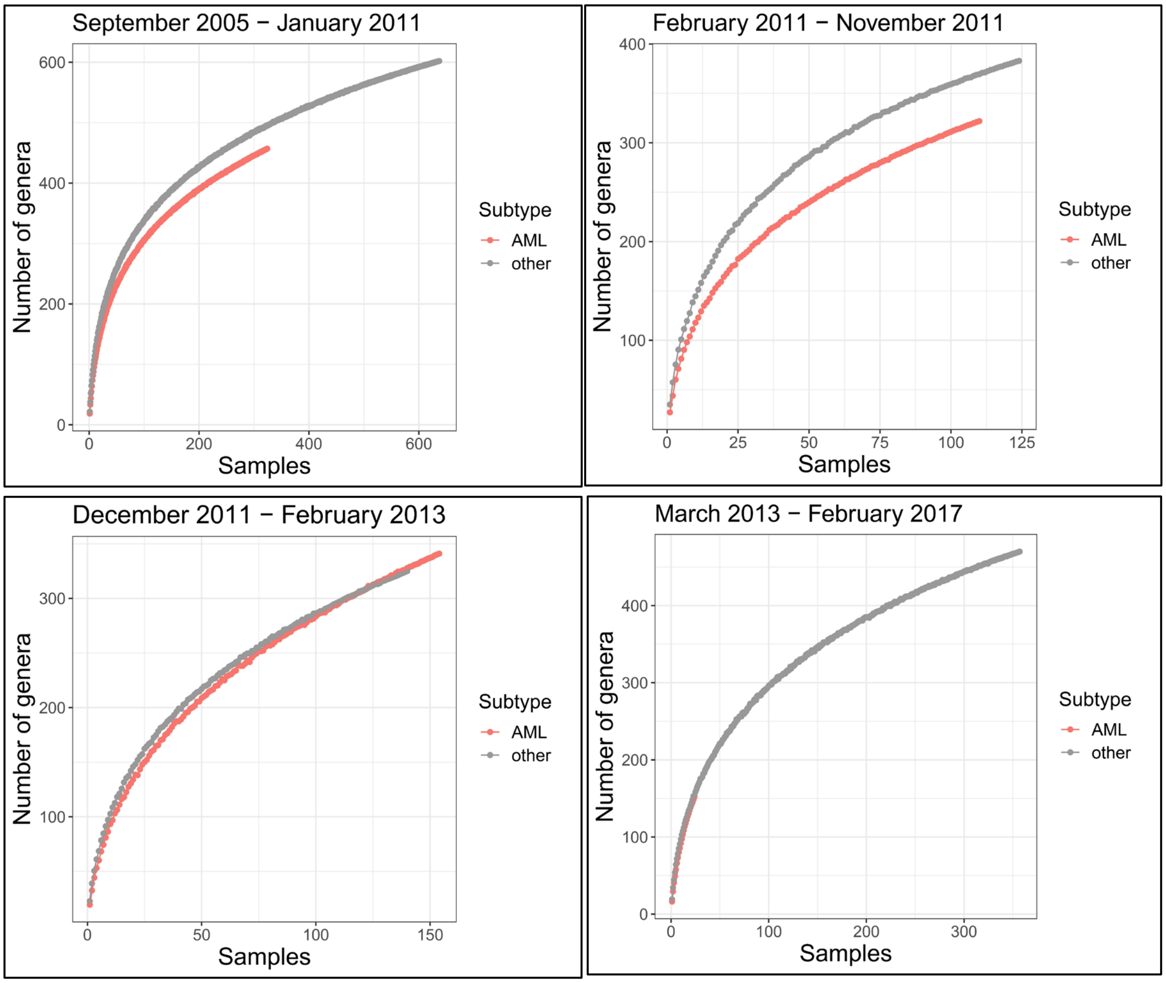


**Supplementary Figure 6:** **Rarefaction plots for AML vs. all other disease subtypes, within each time period separately.**

| | AUROC estimate (95% CI) | | | | |
|---|---|---|---|---|---|
| | **Entire cohort** | **Sep 2005-Jan 2011** | **Feb 2011-Nov 2011** | **Dec 2011-Feb 2013** | **Mar 2013-Feb 2017** |
| **AML vs. all** | 0.87 (0.84-0.90) | 0.89 (0.85-0.93) | 0.75 (0.64-0.85) | 0.64 (0.56-0.72) | 0.57 (0.39-0.75) |
| **MDS vs. all** | 0.84 (0.81-0.88) | 0.88 (0.85-0.91) | 0.77 (0.65-0.86) | 0.70 (0.55-0.80) | 0.77 (0.65-0.88) |
| **MDS/MPN vs. all** | 0.75 (0.70-0.80) | 0.73 (0.61-0.84) | 0.56 (0.35-0.83) | 0.66 (0.51-0.76) | 0.62 (0.53-0.70) |
| **MPN vs. all** | 0.79 (0.75-0.83) | 0.77 (0.70-0.84) | 0.78 (0.61-0.91) | 0.58 (0.42-0.77) | 0.67 (0.59-0.74) |
| **four-way classifier** | 0.84 (0.84-0.84) | 0.83 (0.82-0.83) | 0.71 (0.70-0.73) | 0.64 (0.61-0.65) | 0.61 (0.58-0.63) |

**Supplementary Table 1:** **AUROC estimates for all five disease subtype classifiers, both overall and within each time period.**

## Relationships between blast percentage and microbial features

In the main text, we report associations between patient blast percentage and microbial features: viral detection, viral burden, Proteobacteria relative abundance, and α-diversity. We recomputed the significance of each of these associations by using regression models incorporating time period as above. Supplementary Table 2 below shows the *P*-values and directions of these associations, both adjusted and unadjusted for time period.

**Associations between blast % and microbial characteristics : P-value (direction of association)**

| | unadjusted for time period | adjusted for time period |
|---|---|---|
| viral read presence (logistic regression) | 0.0017 (-) | 0.0021 (-) |
| viral burden (linear regression) | 0.0012 (-) | 0.0012 (-) |
| proteobacteria relative abundance | $1.0 \times 10^{-10}$ (+) | $2.7 \times 10^{-6}$ (+) |
| phylum α-diversity (linear regression) | $< 2.2 \times 10^{-16}$ (-) | $1.5 \times 10^{-11}$ (-) |
| class α-diversity (linear regression) | 0.018 (-) | 0.014 (+) |
| order α-diversity (linear regression) | $3.7 \times 10^{-8}$ (-) | 0.62 (-) |
| family α-diversity (linear regression) | $5.1 \times 10^{-12}$ (-) | 0.0031 (-) |
| genus α-diversity (linear regression) | 0.45 (+) | 0.12 (+) |
| species α-diversity (linear regression) | 0.02 (-) | 0.40 (+) |

(+) positive correlation, (-) negative correlation

**Supplementary Table 2: P-values for associations between blast percentage and microbial characteristics, both adjusted and unadjusted for time period.**

## Relationships between bacteriome and host mutations

Using logistic regression models, in the main text we report significant associations between *DNMT3A* mutations and genus α-diversity, as well as between *FLT3* and *NPM1* mutations and Proteobacteria levels. Incorporating time period terms into the model as above, the associations remain significant (*P* = 0.00018, 0.00062, and 0.019, respectively), and in the same direction as the original associations.

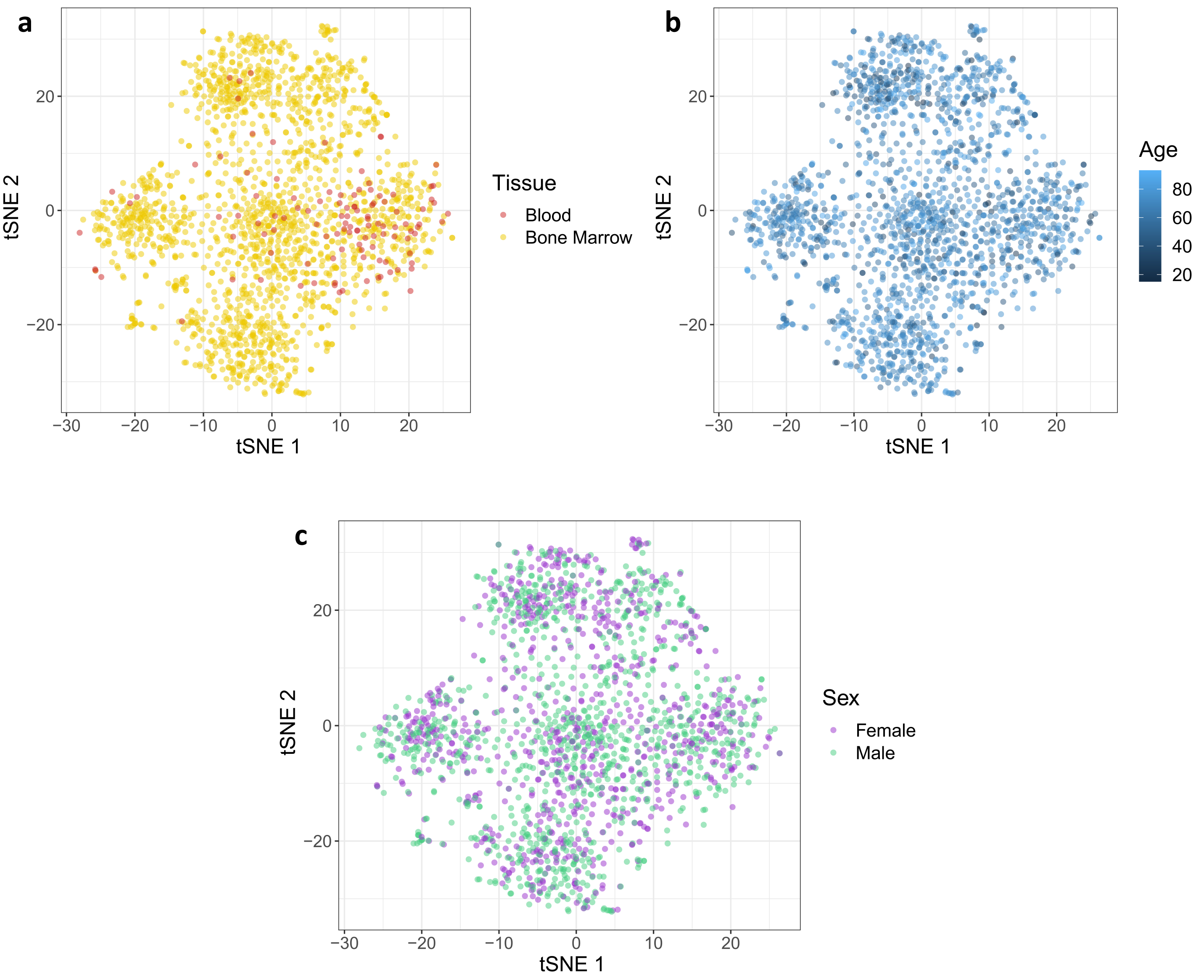


**<u>Supplementary Figure 7:</u> t-SNE plots colored by sample characteristics.** 1870 cases and 12 controls colored by a) blood/bone marrow status, b) age, and c) sex.

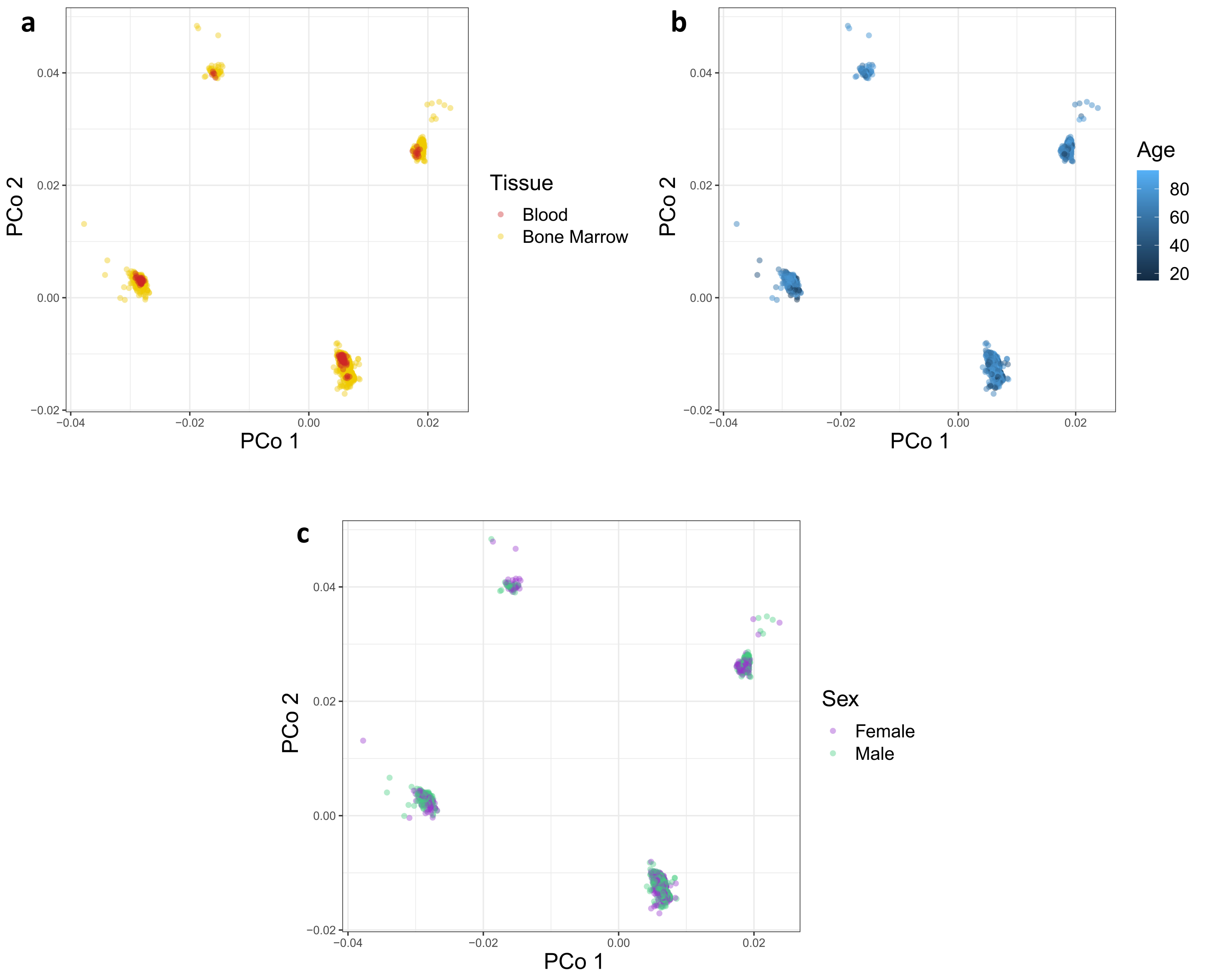


**<u>Supplementary Figure 8:</u> Principal coordinate plots colored by sample characteristics.** 1870 cases colored by a) blood/bone marrow status, b) age, and c) sex. For clarity, two outliers are not shown (bone marrow from males, ages 48 and 81).

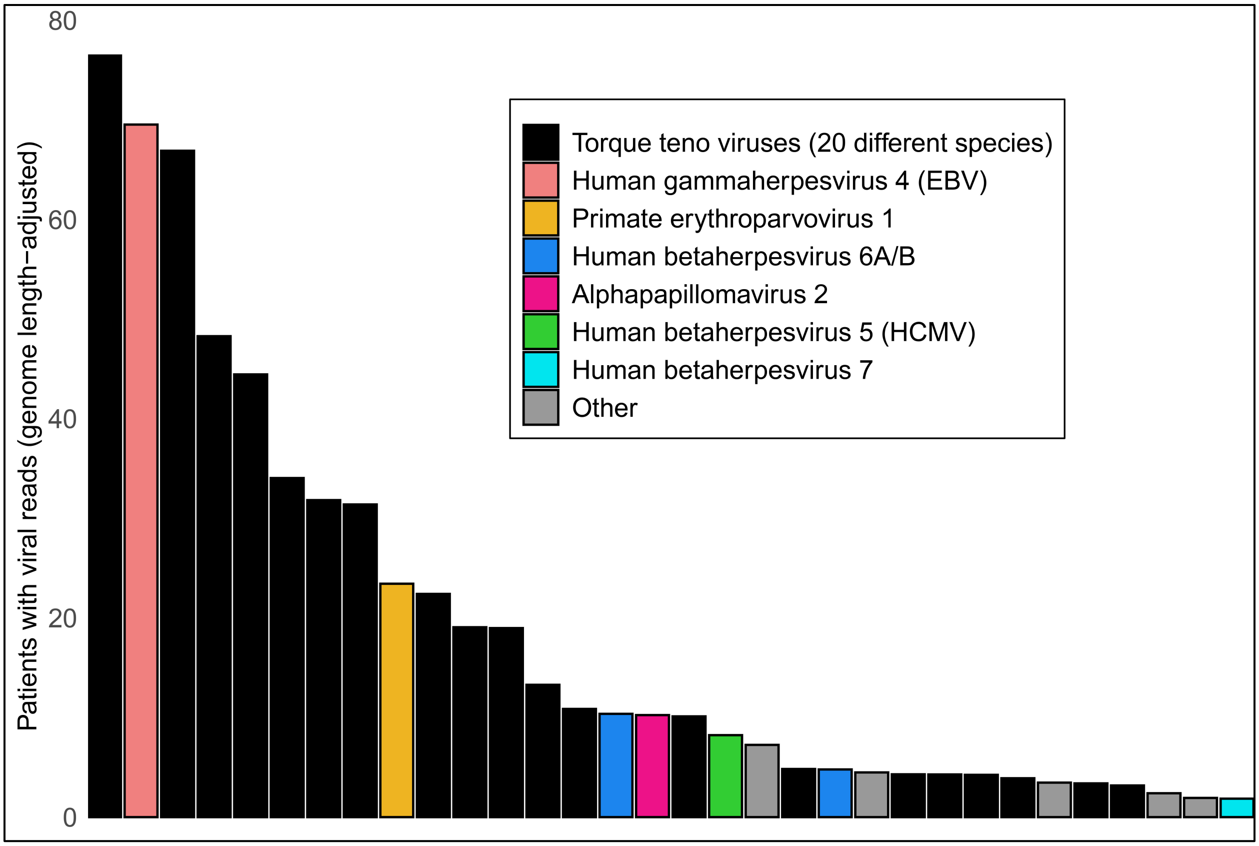


**<u>Supplementary Figure 9:</u> Genome-length-adjusted viral prevalence.** Estimated number of patients each viral species would have been observed in had each virus had the same length (2442 bp).

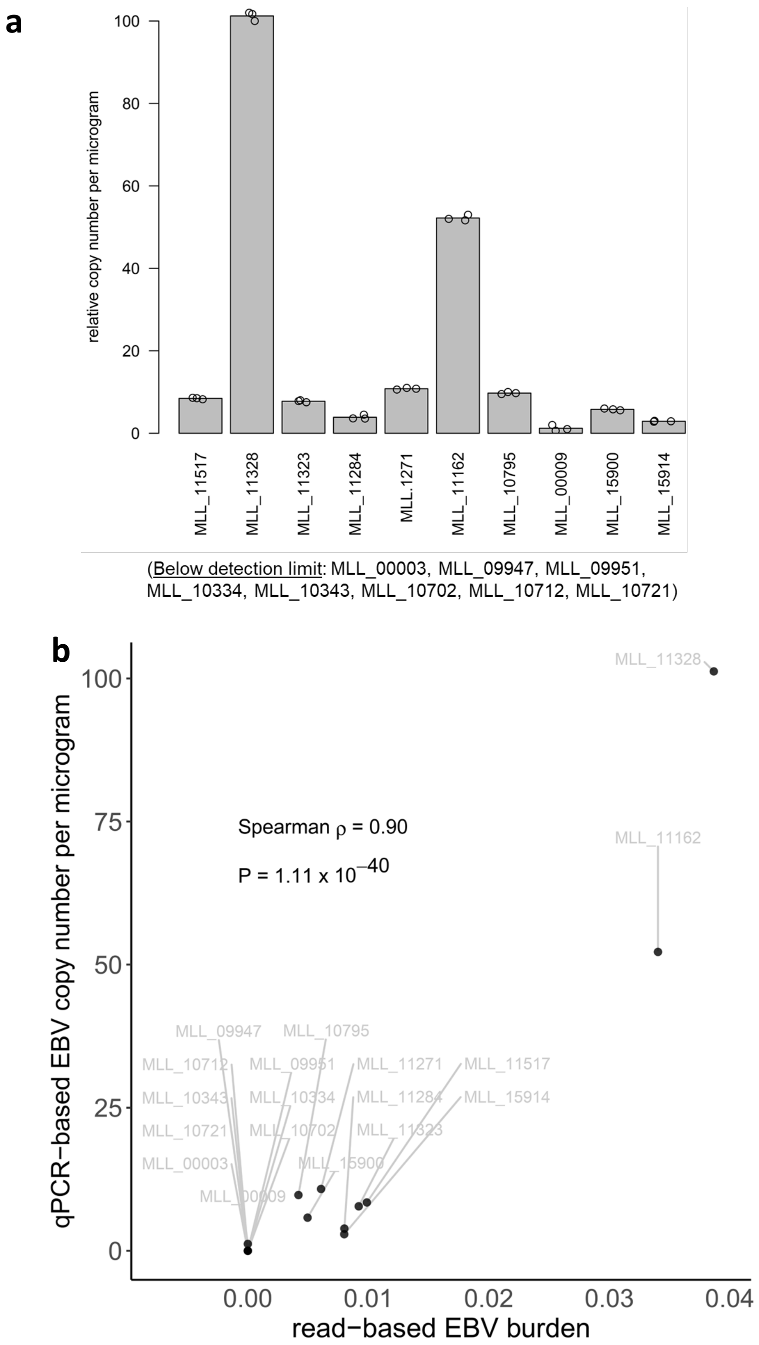


**__Supplementary Figure 10:__ Concordance between read-depth-based and qPCR-based EBV abundance estimated.** 18 DNA samples from our patient cohort were analyzed for EBV presence and abundance using qPCR. a) qPCR EBV copy number estimates over *n* = 3 independent experiments per sample. All three measurements for each sample are shown, and bar height indicates mean of the three experiments. b) Of the 18 samples, nine were deemed EBV-negative according to read-depths because no EBV reads were observed. Of these (lower left corner of plot), eight were also deemed negative based on qPCR, whereas the ninth (MLL_00009) was only barely detectable via qPCR. For the remaining nine samples, both quantification methods were concordant with regard to which samples were outliers (MLL_11162 and MLL_11328) with regard to EBV levels, and which had intermediary levels. Two-sided P-value is computed using cor.test in R with method=”spearman”.
