# Supplementary material for: Circulating microbial content in myeloid malignancy patients is associated with disease subtypes and patient outcomes
Source: Nat Commun. 2022 Feb 24;13:1038. doi: 10.1038/s41467-022-28678-x (PMC8873459; doi:10.1038/s41467-022-28678-x)
Supplement: Supplementary file 3 — Description of Additional Supplementary Files [file 41467_2022_28678_MOESM3_ESM.pdf]

Description of Additional Supplementary Files

Supplementary Data 1: Sample-level read depths

Supplementary Data 2: Taxa removed from consideration

Supplementary Data 3: Putative EBV integration sites
