# Supplementary material for: Circulating microbial content in myeloid malignancy patients is associated with disease subtypes and patient outcomes
Source: Nat Commun. 2022 Feb 24;13:1038. doi: 10.1038/s41467-022-28678-x (PMC8873459; doi:10.1038/s41467-022-28678-x)
Supplement: Supplementary file 7 — Reporting Summary [file 41467_2022_28678_MOESM7_ESM.pdf]

nature research

Corresponding author(s): Thomas LaFramboise

Last updated by author(s): December 31, 2021

Reporting Summary

Nature Research wishes to improve the reproducibility of the work that we publish. This form provides structure for consistency and transparency in reporting. For further information on Nature Research policies, see our Editorial Policies and the Editorial Policy Checklist.

Statistics

For all statistical analyses, confirm that the following items are present in the figure legend, table legend, main text, or Methods section.

n/a	Confirmed	
☐	☒	The exact sample size (n) for each experimental group/condition, given as a discrete number and unit of measurement
☒	☐	A statement on whether measurements were taken from distinct samples or whether the same sample was measured repeatedly
☐	☒	The statistical test(s) used AND whether they are one- or two-sided<br>*Only common tests should be described solely by name; describe more complex techniques in the Methods section.*
☐	☒	A description of all covariates tested
☐	☒	A description of any assumptions or corrections, such as tests of normality and adjustment for multiple comparisons
☐	☒	A full description of the statistical parameters including central tendency (e.g. means) or other basic estimates (e.g. regression coefficient) AND variation (e.g. standard deviation) or associated estimates of uncertainty (e.g. confidence intervals)
☐	☒	For null hypothesis testing, the test statistic (e.g. F, t, r) with confidence intervals, effect sizes, degrees of freedom and P value noted<br>*Give P values as exact values whenever suitable.*
☒	☐	For Bayesian analysis, information on the choice of priors and Markov chain Monte Carlo settings
☒	☐	For hierarchical and complex designs, identification of the appropriate level for tests and full reporting of outcomes
☐	☒	Estimates of effect sizes (e.g. Cohen's d, Pearson's r), indicating how they were calculated

Our web collection on statistics for biologists contains articles on many of the points above.

Software and code

Policy information about availability of computer code

Data collection	No software was used to collect the data.
Data analysis	Illumina BaseSpace whole genome sequencing app version 5.0.0, GATK version 4.0.6.0, bwa version 0.7.17-r1188, R version 4.0.3, R packages survival version 3.2-7, survminer version 0.4.8, randomForest version 4.6-14, PRROC version 1.3.1, pROC version 1.18.0, and ROCR version v1.0-11.

For manuscripts utilizing custom algorithms or software that are central to the research but not yet described in published literature, software must be made available to editors and reviewers. We strongly encourage code deposition in a community repository (e.g. GitHub). See the Nature Research guidelines for submitting code & software for further information.

Data

Policy information about availability of data

All manuscripts must include a data availability statement. This statement should provide the following information, where applicable:

- Accession codes, unique identifiers, or web links for publicly available datasets
- A list of figures that have associated raw data
- A description of any restrictions on data availability

Bam files generated in this study containing all non-human reads have been deposited in the NCBI's database under accession code PRJNA746290 [https://www.ncbi.nlm.nih.gov/bioproject/PRJNA746290]

Human genome build hg19 can be downloaded from https://hgdownload.soe.ucsc.edu/goldenPath/hg19/bigZips/hg19.fa.gz

Field-specific reporting

Please select the one below that is the best fit for your research. If you are not sure, read the appropriate sections before making your selection.

☒ Life sciences ☐ Behavioural & social sciences ☐ Ecological, evolutionary & environmental sciences

For a reference copy of the document with all sections, see nature.com/documents/nr-reporting-summary-flat.pdf

Life sciences study design

All studies must disclose on these points even when the disclosure is negative.

Sample size	The sample size of the study was 1,870 patients with myeloid malignancy. Ours is the first study to analyze the circulating microbiome of this patient population. It is therefore difficult to perform sample size calculations, as the expected dysbiosis and effect sizes were not known a priori. We therefore acquired all of the available patient myeloid malignancy samples. The sufficiency of the sample size is evidenced by the strongly significant statistical results. For the normal samples, we were restricted to the small number (12) of bone marrow donors. Although this sample size left us underpowered to perform comprehensive analyses, we were able to detect statistically significant differences between normal controls and disease cases for some microbiome characteristics.
Data exclusions	DNA sequence reads from known artifactual genera and species were excluded from downstream analysis. These reads were excluded from all results described in the manuscript, and were partially predetermined by existing literature and partially discovered as artifacts during the data analysis steps.
Replication	The RT-qPCR experiments were performed in triplicate for each sample, as independent experiments. All attempts at replication were successful.
Randomization	Randomization was not relevant to this study, as the study's goal was to compare microbiome content between cases and controls, and among diagnoses. Age, sex, diagnosis, and disease status were controlled for.
Blinding	Blinding is not relevant to our study, as our goal was to find microbial differences among groups, therefore necessitating knowledge of group allocation.

Reporting for specific materials, systems and methods

We require information from authors about some types of materials, experimental systems and methods used in many studies. Here, indicate whether each material, system or method listed is relevant to your study. If you are not sure if a list item applies to your research, read the appropriate section before selecting a response.

Materials & experimental systems

n/a	Involved in the study	
☒	☐	Antibodies
☒	☐	Eukaryotic cell lines
☒	☐	Palaeontology and archaeology
☒	☐	Animals and other organisms
☐	☒	Human research participants
☒	☐	Clinical data
☒	☐	Dual use research of concern

Methods

n/a	Involved in the study	
☒	☐	ChIP-seq
☒	☐	Flow cytometry
☒	☐	MRI-based neuroimaging

Human research participants

Policy information about studies involving human research participants

Population characteristics	The study population included myeloid malignancy patients, of which 612 had acute myeloid malignancy, 640 had myelodysplastic syndrome (MDS), 354 had myeloproliferative neoplasia (MPN), and 264 had MDS/MPN overlap syndrome. Overall, 42.2% of patients were female and the median age was 67.1 years with interquartile range 60.9-77.1. The study population also included healthy controls, of which 25% were female and the median age was 44 with interquartile range 27-51.
Recruitment	The patient cohort comprised patients diagnosed with myeloid malignancy whose bone marrow or peripheral blood was sent to the Munich Leukemia Laboratory for diagnostic workup between 2005 and 2017. Given the nature of our study, there is no obvious way that the recruiting procedure would bias the results.
Ethics oversight	The study has been approved by the Internal Review Board of the Munich Leukemia Laboratory as well as by the ethics committee of the Bavarian physicians´ chamber.

Note that full information on the approval of the study protocol must also be provided in the manuscript.
